# Assessing whether universal coverage with insecticide-treated nets has been achieved: is the right indicator being used?

**DOI:** 10.1186/s12936-018-2505-0

**Published:** 2018-10-11

**Authors:** Hannah Koenker, Fred Arnold, Fatou Ba, Moustapha Cisse, Lamine Diouf, Erin Eckert, Marcy Erskine, Lia Florey, Megan Fotheringham, Lilia Gerberg, Christian Lengeler, Matthew Lynch, Abraham Mnzava, Susann Nasr, Médoune Ndiop, Stephen Poyer, Melanie Renshaw, Estifanos Shargie, Cameron Taylor, Julie Thwing, Suzanne Van Hulle, Yazoumé Ye, Josh Yukich, Albert Kilian

**Affiliations:** 1 PMI Vectorworks Project, Johns Hopkins Bloomberg School of Public Health Center for Communication Programs, Baltimore, MD USA; 2 ICF, Rockville, MD USA; 3 National Malaria Control Programme, Ministry of Health, Dakar, Senegal; 4 U.S. President’s Malaria Initiative, U.S. Agency for International Development, Washington, DC USA; 5 International Federation of the Red Cross and Red Crescent Societies, Geneva, Switzerland; 6 Swiss Tropical and Public Health Institute, Basel, Switzerland; 7 University of Basel, Basel, Switzerland; 8 African Leaders’ Malaria Alliance, Arusha, Tanzania; 9 The Global Fund to Fight AIDS, Tuberculosis, and Malaria, Geneva, Switzerland; 10 Population Services International, Washington, DC USA; 11 African Leaders’ Malaria Alliance, Nairobi, Kenya; 12 U.S. President’s Malaria Initiative, U.S. Centers for Disease Control and Prevention, Atlanta, GA USA; 13 Catholic Relief Services, Baltimore, MD USA; 14 PMI VectorWorks Project, Centre for Applied Malaria Research, Tulane University School of Public Health, New Orleans, LA USA; 15 PMI VectorWorks Project, Tropical Health LLP, Montagut, Spain

**Keywords:** ITN, Access, Universal coverage, Bed net coverage, Mosquito net

## Abstract

**Background/methods:**

Insecticide-treated nets (ITNs) are the primary tool for malaria vector control in sub-Saharan Africa, and have been responsible for an estimated two-thirds of the reduction in the global burden of malaria in recent years. While the ultimate goal is high levels of ITN use to confer protection against infected mosquitoes, it is widely accepted that ITN use must be understood in the context of ITN availability. However, despite nearly a decade of universal coverage campaigns, no country has achieved a measured level of 80% of households owning 1 ITN for 2 people in a national survey. Eighty-six public datasets from 33 countries in sub-Saharan Africa (2005–2017) were used to explore the causes of failure to achieve universal coverage at the household level, understand the relationships between the various ITN indicators, and further define their respective programmatic utility.

**Results:**

The proportion of households owning 1 ITN for 2 people did not exceed 60% at the national level in any survey, except in Uganda’s 2014 Malaria Indicator Survey (MIS). At 80% population ITN access, the expected proportion of households with 1 ITN for 2 people is only 60% (p = 0.003 *R*^2^ = 0.92), because individuals in households with some but not enough ITNs are captured as having access, but the household does not qualify as having 1 ITN for 2 people. Among households with 7–9 people, mean population ITN access was 41.0% (95% CI 36.5–45.6), whereas only 6.2% (95% CI 4.0–8.3) of these same households owned at least 1 ITN for 2 people. On average, 60% of the individual protection measured by the population access indicator is obscured when focus is put on the household “universal coverage” indicator. The practice of limiting households to a maximum number of ITNs in mass campaigns severely restricts the ability of large households to obtain enough ITNs for their entire family.

**Conclusions:**

The two household-level indicators—one representing minimal coverage, the other only ‘universal’ coverage—provide an incomplete and potentially misleading picture of personal protection and the success of an ITN distribution programme. Under current ITN distribution strategies, the global malaria community cannot expect countries to reach 80% of households owning 1 ITN for 2 people at a national level. When programmes assess the success of ITN distribution activities, population access to ITNs should be considered as the better indicator of “universal coverage,” because it is based on people as the unit of analysis.

**Electronic supplementary material:**

The online version of this article (10.1186/s12936-018-2505-0) contains supplementary material, which is available to authorized users.

## Background

Insecticide-treated nets (ITNs), which today are almost exclusively comprised of long-lasting insecticidal nets (LLINs), are the primary tool for malaria vector control in sub-Saharan Africa, and have been responsible for an estimated two-thirds of the reduction in the global burden of malaria in recent years [1]. Over 1.5 billion ITNs have been distributed since the UN Secretary General called for a scale-up of ITN coverage in 2008, primarily through mass campaigns aiming at reaching universal coverage [2]. In line with the definition of “universal health coverage”, the World Health Organization (WHO), in the 2017 update of its recommendations on achieving universal coverage with LLINs, defines “universal coverage” as “universal access to, and use of, LLINs” for the entire population at risk of malaria targeted in the control or elimination strategy [3]. Usually the minimum target for universal coverage to be considered achieved is 80% both for access (ownership) and use. This definition of “universal coverage with ITNs” is not disputed, and there is general agreement how to quantify the need for ITNs.

Universal coverage campaigns use an algorithm to calculate the number of ITNs needed for procurement based on population. The definition applies the observation that on average two people share a net, meaning that if one net is given for every two people in a household, all members have a chance to use an ITN [4]. For quantification of the number of ITNs needed for a national mass distribution campaign, the population is divided by 1.8 (and not by 2), to account for households with an odd number of members in which an additional net would be needed [3, 5]. Campaigns also tend not to count existing ITNs in the household, as these are most often older ITNs, with a limited useful life, and the effort of counting and including these in distribution planning would be intensive, and possibly of limited utility [6].

The 2013 revision of malaria indicators by the Roll Back Malaria Monitoring and Evaluation Reference Group [7] recommended four indicators for measuring ITN availability and use (Table 1). Two are calculated at the household level, and two are calculated at the individual (population) level. The two household level indicators are (i) the proportion of households that own at least 1 ITN and (ii) the proportion of households that own at least 1 ITN for 2 people. The two population-level indicators are (iii) the proportion of the population with access to an ITN within the household and (iv) the proportion of the population that used an ITN the previous night. While the ultimate goal is of course high levels of ITN use to confer protection against infected mosquitoes, it is widely accepted that ITN use must be understood in the context of ITN availability [8–11]. Ownership and access indicators are, therefore, useful for malaria programmes to understand the reach and breadth of their ITN distribution activities.

**Table 1:** ITN indicators: advantages and limitations

Indicator	Unit of analysis	What it measures	Advantages	Limitations
% HHs owning at least 1 ITN	Household	Measures what proportion of households has 1 (or more) ITNs	Demonstrates the basic ‘reach’ of ITN distribution activities	Does not indicate the extent to which individuals have the opportunity to use an ITN—as 1 ITN is nearly always insufficient to enable ITN use by all household members
% HHs owning at least 1 ITN for 2 people	Household	Measures what proportion of households has enough ITNs to protect all individuals in the household assuming 2 persons use each ITN	Correlates with WHO and mass campaigns goals of providing 1 ITN for every 2 people; easy to communicate with a broader audience	Underestimates coverage by totally ignoring households that have ITNs to cover a significant portion, but not all, of the individuals in the household
% population that used an ITN the night before the survey	People	Measures the level of ITN use of all age groups at the time of the survey	Provides an exact picture of what proportion of the population is individually protected by an ITN the night before the survey	Low ITN use often assumed to be a behavioural problem, but use is highly driven by ITN access, which is not accounted for by this indicator
% population with access to an ITN within the households	People	Provides an estimate of the proportion of the total population that could have slept under an ITN. Assuming two people share one ITN	Accounts for ITNs in all households and precisely counts all individuals that could use an ITN. Can be directly compared with ITN use to identify specific behavioural gaps	Can be challenging to conceptualize

**Fig. 1:**
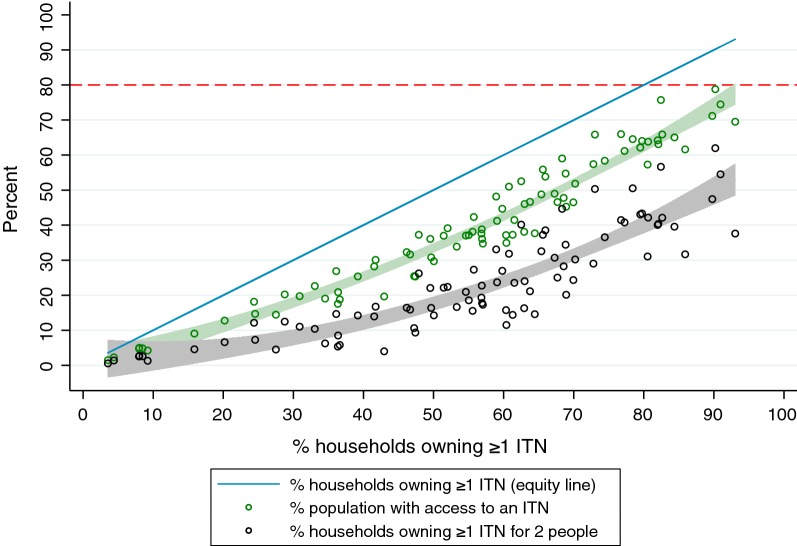
National level population access to ITN (green) and household ownership of ≥ 1 ITN for 2 people (black) plotted against household ownership of ≥ 1 ITN from 86 surveys. Shaded area = 95% CI of fitted values)

The three indicators measuring ownership and access provide different viewpoints into ITN ‘coverage’. Previous work has shown they are mathematically related [6, 11]; the present work focuses on the programmatic utility of the indicators. The proportion of households that own at least 1 ITN provides a sense of the spatial reach of ITN distribution activities, at a minimal depth of coverage. The proportion of households that own at least 1 ITN for every 2 household members is often referred to as “universal coverage”, and may be reworded as the proportion of households with ‘enough’ or ‘sufficient’ ITNs. This seems intuitively to be the best choice for a summary indicator as the algorithm for allocating nets to households to achieve universal coverage and it is also part of the definition of the indicator. Most of the time, country programmes set targets for all ITN indicators at 80% or above in line with WHO recommendations. However, despite 7 years of universal coverage campaigns, no country has achieved a measured level of 80% of households with 1 ITN for 2 people [12] in a national survey, even immediately after a universal coverage distribution campaign. National-level results for the proportion of households owning at least 1 ITN for every 2 people consistently lag behind results for the third indicator, the proportion of the population with access to an ITN within the household (Fig. 1) [12]. This third indicator of ‘coverage’, often referred to as ‘population ITN access’ or ‘ITN access’, provides a population level estimate of individuals who could use an ITN, again based on the assumption that two people can share a net. It is calculated by multiplying the number of ITNs owned by the household by 2, creating a number of ‘potential ITN users’ in the household. Then the number of potential ITN users is divided by the number of household members who stayed in the house the night before the survey (de jure members). Values over 1.00 are set to 1.00, as households cannot have more than 100% access. Each member of the household is then assigned that value in the household member dataset, and the mean is calculated across the population [7, 9]. In the 2017 World Malaria Report, the proportion of households owning any ITN was modelled at 79.7%, population ITN access at 61.2%, population ITN use at 54.1%, and proportion of households that own at least 1 ITN for 2 people in last position at 43.4%.

The objective of this study is to explore reasons why the indicator “the proportion of households owning at least 1 ITN for 2 people” falls consistently far below target levels, and to evaluate the three ITN ownership coverage indicators to see which one is the right indicator to assess whether universal coverage has been achieved.

## Methods

Eighty-six publicly available datasets from Demographic and Health Surveys (DHS) and Malaria Indicator Surveys (MIS) in 33 countries in sub-Saharan Africa (2005–2017) representing all available data sets for this period were downloaded with permission from dhsprogram.com. The proportion of households owning at least 1 ITN for 2 people (enough ITNs) and population access to an ITN within the household were calculated at the national level, according to standard RBM Monitoring and Evaluation Reference Group procedures [7]. Mean household size was calculated using *de jure* (usual) members. All data preparations and analyses were done using Stata 14 software (Stata Corp, College Station, Texas, USA) and applying the sampling weights as provided in the data sets.

To provide data points at the higher end of the range of access and ownership, 12 surveys from eight countries were identified where national-level ownership of at least 1 ITN within the household exceeded 80%. A total of 121 regional or provincial estimates of the same indicators were calculated for these 12 surveys. For 59 surveys conducted in 2010–2016, ITN indicators were calculated by household size (a categorical variable of 1–3, 4–6, 7–10, and ≥ 11 de jure members) and extracted with their standard errors and confidence intervals adjusting for the cluster sampling design.

Country results for all described variables were then extracted into a new data set that also included country and region (where applicable), year and type of survey (MIS, DHS). Multivariable regression analysis was then used to analyse trends and relationships within this data set either as linear regression models or fractional polynomial models, using Stata’s fp command to identify the best fit. Analytical weights were created and applied to adjust for differences in size and variation of data sets. Unless otherwise indicated, statistical significance testing applied the Pearson design-based F-statistic for proportions and ordinary least squares linear regression for multivariable analysis.

## Results

In a simple plot of the national results for each of the three ITN coverage indicators plotted against household ownership of at least 1 ITN, it is observed that household ownership of at least 1 ITN for 2 people is consistently below population ITN access, which itself falls consistently below household ownership of at least 1 ITN (Fig. 1).

**Fig. 2:**
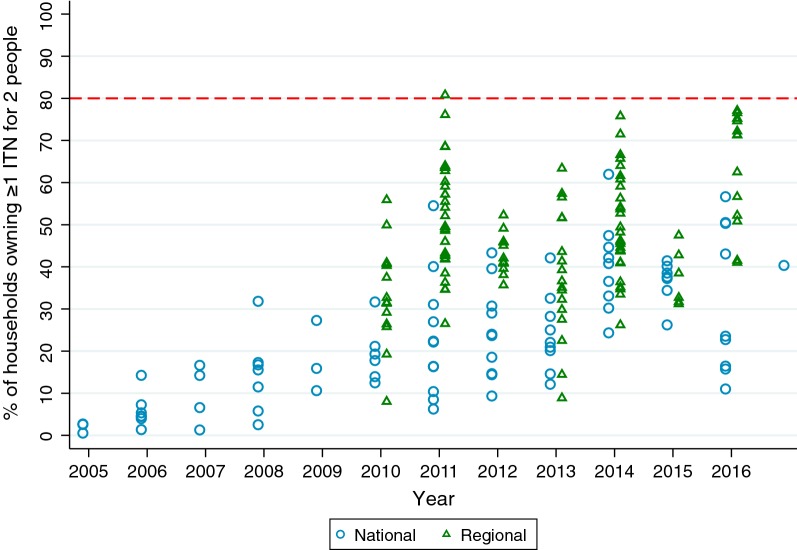
National (blue circles, from 86 surveys) and selected regional estimates (green triangles, from 12 surveys where national-level ownership of at least 1 ITN was > 80%; offset for readability) for household ownership of at least 1 ITN for every 2 people, 2005–2016. Target line of 80% indicated in dashed red

Even immediately following universal coverage campaigns, the highest result for the proportion of households owning at least 1 ITN for 2 people was only 62.0% at the national level, in Uganda’s 2014 MIS. Among the regional data points, the highest result was 81.0% in Lindi, Tanzania, in the 2011–2012 Tanzania HIV/AIDS and Malaria Indicator Survey (Fig. 2).

In the 12 surveys where household ownership of at least 1 ITN exceeded 80%, population ITN access ranged from 57.3% in Madagascar 2011 to 78.8% in Uganda 2014 (Table 2). In these same surveys, the proportion of households owning at least 1 ITN for 2 people ranged from 31.1% in Madagascar 2011 to 62.0% in Uganda 2014.

**Table 2:** ITN indicators in household surveys conducted shortly after universal coverage campaigns

Country	Survey	Year	% households owning ≥ 1 ITN	% households owning ≥ 1 ITN per 2 people	% population with access to an ITN	Mean household size (de jure)
Madagascar	MIS	2011	80.5	31.1	57.3	4.9
Rwanda	DHS	2014	80.6	42.2	63.8	4.9
Benin	DHS	2012	81.8	43.3	64.0	5.0
Rwanda	DHS	2010	82.0	40.7	64.2	4.4
Senegal	DHS	2016	82.1	57.1	75.8	8.3
Rwanda	MIS	2013	82.6	42.1	65.9	4.3
Mali	DHS	2013	84.4	39.6	65.1	5.7
Mali	AP	2010	85.9	31.7	61.6	6.0
Burkina Faso	DHS	2014	89.8	47.4	71.2	5.9
Uganda	MIS	2014	90.2	62.0	78.8	4.9
Tanzania	MIS	2011	91.0	54.8	74.7	5.1
Mali	MIS	2015	93.0	37.6	69.5	9.0

**Fig. 3:**
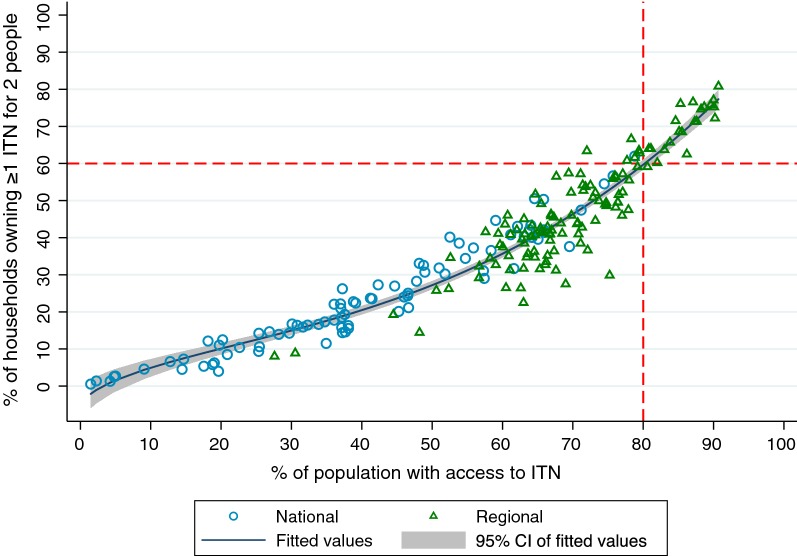
Household ownership of ≥ 1 ITN for 2 people vs population ITN access with national and regional results. Fitted values use a best-fit fractional polynomial

Population ITN access and household ownership of sufficient ITNs were highly correlated, with household ownership of 1 ITN for 2 people on average estimated to be 67.0% that of population ITN access (coef. 0.67 95% CI 0.65–0.69; p < 0.0001; R^2^ = 0.97) in a linear model when the no-constant option was specified to force the intercept at zero for the two indicators. However, as shown in Fig. 3, the fractional polynomial model was a better fit than the linear model (p = 0.003), demonstrating that at 80% population ITN access, the proportion of households owning ≥ 1 ITN for 2 people is estimated to be approximately 60%. Models for each indicator are presented in Additional file 1.

**Fig. 4:**
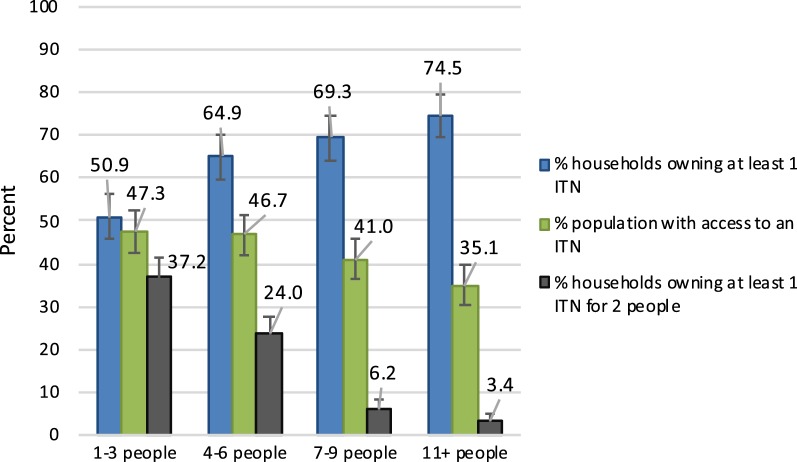
Mean ITN coverage for the three ITN coverage indicators for households of varying sizes, across 59 household surveys (2010–2016)

Household size was a key determinant of all three ITN coverage indicators. Among surveys conducted in 2010–2016, ownership of at least 1 ITN increased as household size increased (coef. = 1.36; p < 0.001). However, increasing household size was significantly associated with lower levels of household ownership of at least 1 ITN for 2 people (coef. = − 2.24; p < 0.001), with only 6.2% (95% CI 4.0–8.3) of households of 7–9 people reaching this threshold. Larger households also had reduced levels of population ITN access, but the decline was less pronounced (coef. = − 0.66; p = 0.010). Across the 2010–2016 surveys, population ITN access was 41.0% (95% CI 36.5–45.6) among those in households with 7–9 people, whereas these same households only had rates of 6.2% of owning at least 1 ITN for 2 people (Fig. 4).

**Fig. 5:**
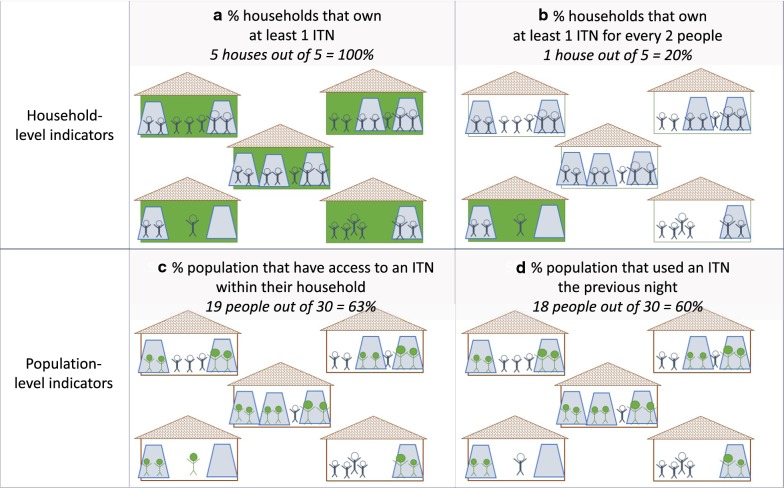
Illustrative depiction of ITN indicators using 5 households, 30 individuals, and 10 ITNs. The top row **a**, **b** demonstrate household ownership indicators, while the bottom row **c**, **d** shows population-level indicators. ITNs are depicted in tall trapezoids and individuals with stick figures. Households meeting the indicator criteria for ownership are identified in green/darker color. Individuals meeting the indicator criteria are identified with solid green color

To provide a simplified illustration of the individual protection that is obscured by the household indicator of owning at least 1 ITN for 2 people, compared to the population access indicator, Fig. 5 depicts five households, in which a total of 30 people reside, with 10 ITNs. All five households own at least 1 ITN (Fig. 5a), but only one (the smallest household) owns at least 1 ITN for every 2 people (green house in Fig. 5b). However, as illustrated with the green stick figures in Fig. 5c, 19 people have access to an ITN within their household, out of 30 (63%). Ultimately, 18 of those 19 individuals with access slept under an ITN the previous night (green figures in Fig. 5d), giving a total population use of 60%.

If one looks at the number of people that are assumed to be protected with the indicator of proportion of households owning at least 1 ITN for 2 people, only three individuals (10%) are counted. Compare this with the 19 people that are counted under the population access indicator. In effect, 16 individuals out of the 30 in this hypothetical village—53%—have access to an ITN that is ignored when looking only at the indicator of households with enough ITNs. This is the same as the crude difference between 63% population ITN access and 10% (3 of 30) population living in households that own enough ITNs. The ignored population can also be expressed as a percentage of population access: 16 individuals out of the 19 individuals with access to an ITN in this village are ignored by the indicator of households that own at least 1 ITN for 2 people, or 84%.

**Fig. 6:**
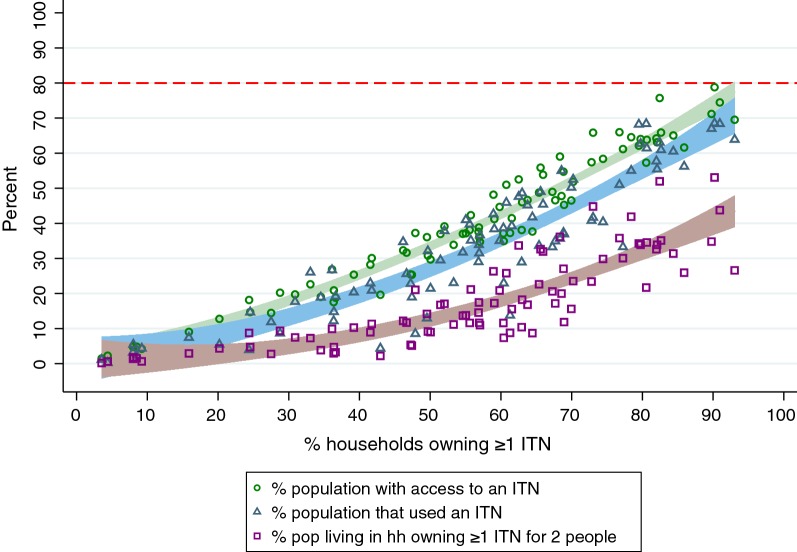
The proportion of people with access to an ITN (green) and the proportion of people living in households with enough ITNs (yellow) as a function of household ownership of any ITN. The difference between the green and yellow plots is 20 percentage points on average, and the gap is on average 60% of the percentage of population ITN access

Expanding this analysis to the 86 datasets (Fig. 6), the average difference between the population with access to an ITN and the population living in households that own enough ITNs is 21%. This is the overall percentage of the population that has access to an ITN within their household that is ignored when looking solely at the indicator of proportion of households that own at least 1 ITN for 2 people. But because this crude difference is smaller at lower levels of population access, and higher at high levels of access, it is better expressed as a percentage. The percentage of population access that is ignored, out of the total population with access, can be expressed as

$$\% population\;whose\;ITN\;access\;is\;ignored\;by\;the\;indicator\;of\; ``\% \;households\;owing\;at\;least\;1\;ITN\;for\;2\;people" = 1 - \left( {\frac{\% \;population\;living\;in\;households\;with\;enough\;ITNs}{\% \;population\;with\;access\;to\;an\;ITN}} \right)$$

On average, across the 86 national surveys reviewed in this study, out of all the people with access to an ITN, 60% (95% CI 23–89%) are ignored when planners focus solely on the indicator of households owning at least 1 ITN for 2 people.

## Discussion

Currently, targets in national strategic plans or donor documents for all three ITN coverage indicators are usually set at 80% or above. This gives a perhaps unintended implication that the three indicators should increase together at the same rates. Moreover, universal coverage guidance from WHO and others calls for procuring ITNs with the goal of providing each household with 1 ITN for 2 people, again implying that by doing so, countries should expect to achieve 100% of households owning enough ITNs immediately after a mass campaign. These implied expectations contribute to confusion and frustration when post-campaign results—particularly for the indicator of households owning at least 1 ITN for 2 people—are far below target levels. This also has significant implications for donor funding. Performance frameworks, typically aligned to the household ownership indicators, may inadvertently set national programmes up for failure, and countries may be unnecessarily penalized based on the indicator targets, not all of which are achievable.

This work builds on previous work describing the utility of the ITN indicators and exploring the mathematical relationships between the indicators in order to model ITN coverage in years between surveys [6, 9, 11]. However, in terms of programmatic utility, each indicator must be considered with its advantages and limitations, in terms of interpreting the extent of ITN protection in a given population. The two household indicators can be compared to provide an ‘ownership gap’, and the two population indicators can be compared to provide a ‘use gap’ [9, 11].

The proportion of households owning at least 1 ITN is a minimal threshold that essentially describes the spatial reach of ITN distribution activities, but not the degree to which the population is protected. (The vast majority (80%) of households in endemic countries require more than a single ITN to protect all persons in the household—see Additional file 2). At the other end of the spectrum, the proportion of households owning at least 1 ITN for 2 people is an indicator of ‘perfect’ household coverage, and has never been reached at a national or even a subregional level. Households may miss qualifying as having ‘enough’ nets by only 1–2 nets, and this is often misinterpreted as these households not having any protection. The two household-level indicators—one representing minimal coverage, the other only ‘universal’ coverage—thus provide an incomplete and potentially misleading picture of personal protection and the success of an ITN distribution programme.

Larger households were far less likely than smaller households to own enough ITNs for all their household members. In fact, many individuals in these households that own some but not enough ITNs had access to a net, and (in most cases) were sleeping under one, as illustrated in Fig. 6. This individual protection is obscured when programme planners focus only on the household-level indicator.

Given these limitations of the household level indicators, the population ITN access indicator is a far better indicator of ‘universal coverage’ because it is based on individual people. It provides a clear picture of the proportion of individuals in a given setting that have the opportunity to use an ITN. It can also be directly compared to the proportion of the population that used an ITN the previous night, which enables detailed analysis of specific behavioral gaps nationally as well as among population subgroups. Ultimately, of course, ITN use is the key behavior required for malaria control, but people cannot use an ITN to which they do not have access. Recent research demonstrates clearly that rates of ITN use among those with access to an ITN with few exceptions are at or above an 80% target [10–13]. Therefore, increasing ITN access will lead directly to increases in ITN use.

It is important to consider and address the programmatic and policy factors that prevent households from obtaining enough ITNs. The primary programmatic reason is that larger households rarely receive the necessary number of ITNs during mass campaigns. During the process of household registration, programmes often put a cap on the number of ITNs any given household can receive to reduce the opportunity for fraud due to inflated numbers of household members. Second, during the process of distribution itself, campaign staff may also pragmatically ration ITNs if they think not enough are available, to remain certain that all households in their catchment area will receive at least some ITNs. Third, larger households, which tend to have more children than smaller households, may have some sleeping spaces in which more than 2 people are sharing an ITN [4] and, therefore, may not require (or be motivated to acquire) ITNs in the 1:2 ratio.

Policy decisions are also likely affecting the ability of large households to receive enough ITNs. The current guidance from the WHO recommends that mass campaigns be planned with a quantification algorithm of “population divided by 1.8,” which is intended to account for the odd-numbered households that require an additional ‘half net’ according to the 1 for 2 ratio. In 2017 the algorithm was updated by the WHO to allow a 10% buffer accounting for outdated census projections [14]. However, when looking more closely at sleeping space patterns, researchers have found reasons to question the current quantification guidance. Analysis of discordant ITN-person pairs (e.g., two roommates in a household who do not share a sleeping space) as well as the trend that with increasing wealth and also increasing ITN access, the number of people per ITN decreases, imply that a more accurate expectation would be that each ITN only protects on average 1.6 people [4]. Whether inaccurate population estimates or an inadequate quantification factor are the greater determinant of ITN gaps during mass campaigns remains to be explored.

Three possible solutions to these supply challenges can be considered. First, if the current levels of ITN access are considered sufficient in epidemiological terms to maintain malaria control, then no changes in distribution strategy are required. Rather, the current targets could be adjusted to be more appropriate for each indicator. Enormous reductions in malaria morbidity and mortality have been observed over the past decade, during which ITN coverage has not been at target levels and yet this ‘insufficient’ ITN coverage has been credited with two-thirds of the observed reductions in morbidity [1]. Modeling work also indicates that community-level protection can be achieved at lower-than-universal coverage levels of 35–65% population use of ITNs [15]. On this basis, the target levels for each ITN coverage indicator might be adjusted to be more pragmatic—i.e. a 95% target for household ownership of any ITN, which corresponds roughly to an 80% target for population ITN access, 70% population ITN use, and a 55% target for household ownership of at least 1 ITN for 2 people.

Second, if current levels of ITN access are not considered sufficient for malaria control, and targets for ITN use should be 80%, as outlined in the WHO Global Technical Strategy (implying a population ITN access target of 90%) [16], then it follows that additional ITNs would need to be procured, potentially using a 10% buffer for mass campaigns, and/or by increasing ITN distribution through ongoing school or community based channels. The WHO currently calls for mass campaigns every 3 years using the population/1.8 quantification factor with an optional 10% buffer, which is equivalent to a population/1.6 quantification. The guidelines further recommend additional ITN distribution as needed to maintain target levels [14], but there is no robust guidance on how many additional ITNs might be needed, nor the most efficient combination of distribution strategies, which inhibits programmes from moving away from triennial mass campaigns. Additional research to provide more specific estimates, including cost-effective options for optimizing ITN coverage over time and space, would likely ease this process for programme planners and donors.

Third, there is some scope for increasing programmatic efficiency in ITN distribution and attempting to use existing quantities of ITNs to achieve higher rates of coverage. This can be done regardless of whether the above two solutions are implemented. First, programmes must acknowledge that large households require more nets, and either avoid setting caps, or set them taking into account regional demographic variations in household sizes. Data on household size are regularly reported in large national surveys such as DHS and MIS, and are summarized here in Additional file 2. Caps currently serve both to limit the negative impact of respondents inflating household size during registration and to ensure that in situations where not enough ITNs are available, all households receive at least a few ITNs. Some regions may indeed have only a small percentage of households that are larger than 8 people; a cap of four ITNs per household might work well. However, in other regions, a cap of four ITNs per household may automatically exclude 15% of households from reaching the target of 1 ITN for 2 people, as in Ghana’s northern regions where the average household size is larger than the rest of the country [17]. Obviously, additional mechanisms to avoid inflation of the numbers of household members during the campaign’s registration phase are and should be put in place. In areas where caps have been used, ‘deflation’ of household size has also been observed—splitting larger households into two or more smaller households to avoid the cap. Second, it has been shown previously that the quality of census and household registration data contribute much more to successful campaigns than other factors [18]. Therefore, investing in household registration and its supervision will help to ensure that all households—whether large, small, or hard to reach—are reached and accurately served. Additional research will be needed to thoroughly assess cost-effective strategies for capping.

There are some minor methodological factors related to achieving universal coverage (based on either indicator) that should be noted. The standard MIS/DHS net roster only lists up to seven ITNs, ignoring any additional nets in the household. In the Mali 2015 MIS, 13% of households owned 7 ITNs, and it is likely that many households own 8 or more nets. These additional nets, however, are not counted and, therefore, these households (if large) may miss reaching the threshold of owning 1 ITN for 2 people solely as a result of this approach. Other countries, including Senegal, have modified the standard net roster to allow for additional ITNs to fit their context. Likewise, the definition of a household—whether for household survey purposes or for mass campaign planning and registration—is certain to be problematic if not done consistently.

## Conclusion

Based on the above findings, the authors recommend that national programmes, donors, and implementing partners focus on the proportion of the population with access to an ITN within the household as the key indicator of universal coverage. Use of this population-level indicator as the primary measure of ITN coverage will strengthen national strategies, implementation plans, policy documents, and DHS, MIS, and MICS reports. The household-level indicator of owning at least 1 ITN for 2 people can be retained, but serve as a secondary indicator. Programmes will need to understand its limitations, and set targets accordingly. Under current ITN quantification and distribution strategies, an 80% target for households owning at least 1 ITN for 2 people is not achievable at a national or even subnational level. This disconnect may inadvertently lead to countries being penalized for continued malaria funding that is contingent upon performance.

This indicator is highly sensitive to average household size, and it masks significant individual protection—on average, 60% of the individual protection measured by the population access indicator is ignored when focus is put on the household “universal coverage” indicator. Given individual ITN use is only possible when a person has access to an ITN within their household, measuring actual ITN access in targeted geographical areas is the more programmatically useful indicator. Population access to ITNs, because it is based on persons as the unit of analysis, should be considered as the primary indicator of ITN coverage when assessing the success of ITN distributions.

## Additional files

**Additional file 1.** Each of the four ITN indicators discussed in this paper are presented plotted against each other. Plots were made using Stata’s “aaplot” function, in which linear (gray lines), quadratic (pink lines), and fractional polynomial fits (shaded gray) can be compared. For each plot the equations for the quadratic and the linear models are listed, with the R^2^ value, describing the proportion of the variance attributable to the included variables. Equations may be useful to inform modeling of these indicators.

**Additional file 2.** For the most recent DHS or MIS survey available in each country, the proportion of households with at least 7, 9, 11, 13, and 15 usual residents was calculated, for each region or province. These represent the proportion of households that would be prevented from receiving 1 ITN for 2 people if caps are set during mass ITN campaigns at 3, 4, 5, 6, or 7 ITNs, respectively. Programme planners may wish to consult these tables when considering setting caps for each region where ITNs will be distributed. This assumes that the definition of a ‘household’ remains the same during the campaign as in the surveys, generally ‘people eating from the same pot’.
