# Supplementary material for: Assessing whether universal coverage with insecticide-treated nets has been achieved: is the right indicator being used?
Source: Malar J. 2018 Oct 11;17:355. doi: 10.1186/s12936-018-2505-0 (PMC6180430; doi:10.1186/s12936-018-2505-0)
Supplement: Supplementary file 1 — Additional file 1. Each of the four ITN indicators discussed in this paper are presented plotted against each other. Plots were made using Stata’s “aaplot” function, in which linear (gray lines), quadratic (pink lines), and fractional polynomial fits (shaded gray) can be compared. For each plot the equations for the quadratic and the linear models are listed, with the R2 value, describing the proportion of the variance attributable to the included variables. Equations may be useful to inform modeling of these indicators. [file 12936_2018_2505_MOESM1_ESM.pdf]

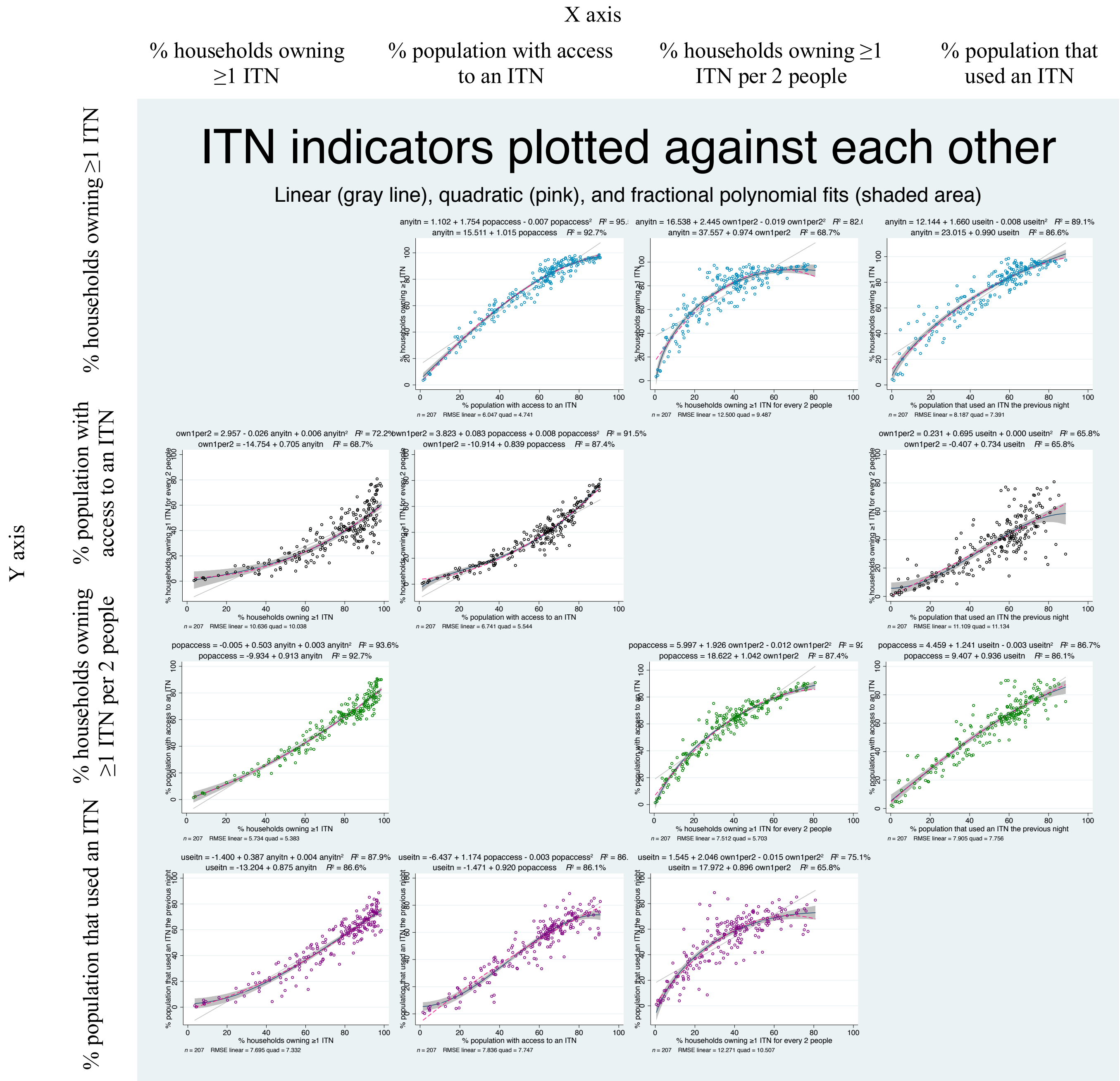
X axis
% households owning ≥1 ITN
% population with access to an ITN
% households owning ≥1 ITN per 2 people
% population that used an ITN
ITN indicators plotted against each other
Linear (gray line), quadratic (pink), and fractional polynomial fits (shaded area)
Y axis
% households owning ≥1 ITN
% population with access to an ITN
% households owning ≥1 ITN per 2 people
% population that used an ITN
